# RGB-D Cameras and Brain–Computer Interfaces for Human Activity Recognition: An Overview

**DOI:** 10.3390/s25206286

**Published:** 2025-10-10

**Authors:** Grazia Iadarola, Alessandro Mengarelli, Sabrina Iarlori, Andrea Monteriù, Susanna Spinsante

**Affiliations:** 1 Department of Information Engineering, Polytechnic University of Marche, 60131 Ancona, Italy; a.mengarelli@staff.univpm.it (A.M.); a.monteriu@staff.univpm.it (A.M.); s.spinsante@staff.univpm.it (S.S.); 2 Department of Theoretical and Applied Sciences (DiSTA), Università degli Studi eCampus, 22060 Novedrate, Italy; sabrina.iarlori@uniecampus.it

**Keywords:** human activity recognition, assisted living, RGB-D cameras, brain–computer interfaces, wearable devices

## Abstract

This paper provides a perspective on the use of RGB-D cameras and non-invasive brain–computer interfaces (BCIs) for human activity recognition (HAR). Then, it explores the potential of integrating both the technologies for active and assisted living. RGB-D cameras can offer monitoring of users in their living environments, preserving their privacy in human activity recognition through depth images and skeleton tracking. Concurrently, non-invasive BCIs can provide access to intent and control of users by decoding neural signals. The synergy between these technologies may allow holistic understanding of both physical context and cognitive state of users, to enhance personalized assistance inside smart homes. The successful deployment in integrating the two technologies needs addressing critical technical hurdles, including computational demands for real-time multi-modal data processing, and user acceptance challenges related to data privacy, security, and BCI illiteracy. Continued interdisciplinary research is essential to realize the full potential of RGB-D cameras and BCIs as AAL solutions, in order to improve the quality of life for independent or impaired people.

## 1. Introduction

Born from the joint use of sensors, communication protocols and artificial intelligence techniques, active and assisted living (AAL) plays a fundamental role in supporting independent and disabled people. AAL is aimed at increasing the autonomy of persons at home, by improving their quality of life or caregiving activities for elderly and impaired people. In such a way, AAL solutions can be beneficial to healthcare in general, by concurring in saving resources through the cost reduction related to institutionalized treatments, and also by easing prevention strategies for chronic diseases. Indeed, pathologies affecting musculoskeletal and neurological systems primarily impact elderly independent life and require resource-intensive care [1]. The potential benefits carried out by AAL systems appear nowadays essential considering the perspectives of developed countries from a demographic point of view. For example, in Europe, the population with 65 years or older will expand significantly, rising from 90.5 million at the start of 2019 to 129.8 million by 2050. Specifically, the cohort aged 75–84 years is projected to increase by 56.1%, while the cohort aged 65–74 years is projected to increase by 16.6% [2]. Furthermore, the social change due to population aging has entailed the demand for assistance to elderly with disabilities to cope with a reduction in social activities and consequent frustration, sadness and depression [3,4].

The need for innovative solutions to AAL challenges to help people pushes towards the development of smart homes that can provide valuable support to maintain an active lifestyle. With the development of more affordable and sophisticated technologies, human activity recognition (HAR) continues to raise great interest among the research community. HAR systems exploit sensors, user-generated datasets and learning algorithms to identify and discriminate everyday activities recurring in people [5,6,7,8], especially elderly or impaired [9,10]. Within this context, AAL technologies found their application in easing the independence of impaired people through the support of daily living activities [11]. Furthermore, they should allow relatives and caregivers to carry out continuous monitoring on the daily habits and to receive prompt warnings in case of falls or possibly risky situations [12]. AAL solutions should also be able to monitor the emotional state and the cognitive deterioration of the person [13]. An additional important aspect is the decoding of the user intention so that the interaction with assistive technologies would be safer and more effective [14]. Finally, the connection between people at home and the health and social services is also of paramount importance [15].

HAR is the object of many research efforts [16], being fundamental for the development of smart home environments [12,17]. In the HAR field, the approach employing vision-based devices is the most common, involving technologies external to body location, such as RGB, video, depth, or thermal cameras. The other possible approach is based on wireless wearable devices, such as sensors for acquisition of bioelectric signals, inertial sensors—inertial measurement units (IMU) and magnetic, angular rate and gravity (MARG) sensors—or brain–computer interfaces (BCI). Both approaches enable non-invasive monitoring of elderly or disabled people, at home or in care facilities [18]. Specifically, in the last fifteen years, among vision-based technologies, the most adopted is represented by RGB-D cameras, while, among wireless wearable technologies, the use of non-invasive BCIs has seen a rapid growth [19]. By collecting neural signals from the user, which can be converted into commands, BCIs enable new approaches to human–device interaction in smart living environments [20]. Their usage can be further advanced by the fusion with information on the performed activity provided by environmental sensors, such as RGB-D cameras.

Generally speaking, given the complexity of human activities, a uni-modal (single) sensing solution may not be enough for their recognition based on machine learning (ML), while multi-modal sensor fusion, enhancing the available features [21], emerges to face critical gaps in AAL technology [22]. Indeed, with multi-modal sensor fusion, combining various sensor types can overcome individual shortcomings, leading to more robust and accurate monitoring and assistance. AAL applications could be designed through RGB-D cameras and non-invasive BCI integration, specifically to improve the environmental perception of actions performed by users.

Differently from existing literature that highlights advantages and limitations of technologies for uni-modal sensing solutions [4,6,10,17,23,24,25,26,27,28,29], this overview is intended to propose a perspective on RGB-D cameras and non-invasive BCIs for HAR, exploring the rationale for the integration of the two technologies and opening a discussion on possible applications that could benefit from such integration. The rest of the paper is organized as follows. Section 2 offers an introduction to current approaches to HAR, while Section 3 and Section 4 provide an overview on RGB-D cameras and non-invasive BCIs, respectively, also focusing on specific techniques based on the use of these two sensing technologies. Potential innovations, but also barriers, stemming from integrated solutions are presented in Section 5. Finally, Section 6 draws the main conclusions.

## 2. Background: Approaches for HAR

**Figure 1:**
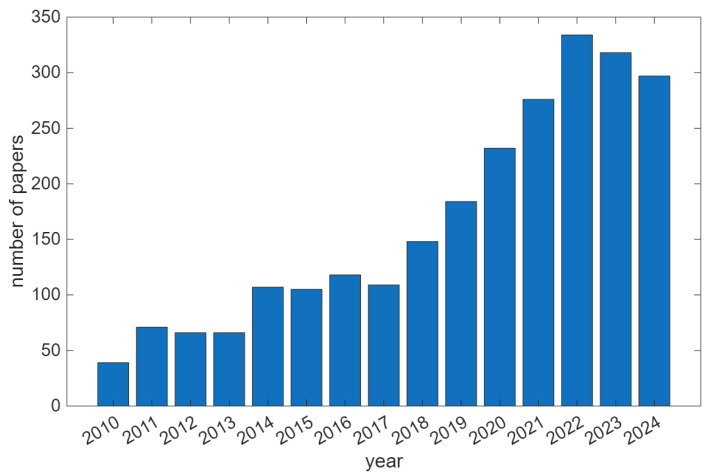
Number of papers related to HAR since 2010. Source: PubMed, June 2025, search query in title/abstract: (activity recognition) OR (HAR).

Figure 1 reports the increasing trend of papers related to HAR in the time range from 2010 to 2024. Among human activities of interest, monitoring of body movements (such as walking or running) represents one of the most investigated aspects for the evaluation of effort and muscle fatigue [8], but applications can include a wider spectrum of physical tasks [25] and activities of daily living (ADLs), namely, hand gestures, sleeping, standing or falling.

Although several human behaviors and activities have been investigated in the field of smart sensing, such as rehabilitation and even human emotional condition by affective computing [24], the outbreak of the COVID-19 pandemic has contributed to stress on two different aspects related to HAR. On the one hand, it put attention on the importance of correctly identifying a series of activities related to human well-being and health, such as those involved with personal hygiene [30]. On the other hand, the pandemic highlighted the value of technical solutions capable of remotely monitoring user activities, habits, and behaviors within a domestic scenario.

As mentioned in the Introduction, two common approaches can be leveraged for HAR within a closed environment, i.e., vision-based devices, such as cameras, and wearable devices. Although vision-based devices represent one of the most common approaches in research, they can mainly lead to two potential drawbacks that hamper their widespread diffusion for HAR purposes. The former is the need for instrumented homes with video-capture sensors to install within each room where the user is supposed to perform the activities of interest. The latter is that vision-based devices record images not only of the living environment but also of subjects, generating several issues regarding privacy and acceptability. Instead, wearable devices can provide information related to the performed activities not directly linked to a specific user and can be processed with a small computational delay, thus potentially allowing also an almost real-time recognition of specific activities performed by the subject. Anyway, also wearable devices, despite their mobility benefits, necessitate consistent user compliance, which can be a significant barrier to long-term monitoring. A review specifically focused on the use of wearable devices for AAL has been proposed in [10]. Table 1 lists both vision-based devices and wearable devices for the main ADLs.

When designing a measurement system that leverages both vision-based and wearable devices for HAR, the aim should be to develop a solution able to correctly identify ADLs with a specific and clear impact on subject well-being and health. Specifically, two main points merit attention. The first point regards the human activities to be recognized by the system and those activities that should be included in the whole set. As outlined above, a wide spectrum of possibilities is available in this case, ranging from activities involving full body movements, such as walking and running, to activities that mainly rely on upper limb motion, including interaction with objects [31]. In fact, the main activities to take into account should be those commonly performed during daily living with upper limbs, such as eating gestures. The rationale for this choice is based on their importance for many aspects related to health and well-being. For instance, a correct identification of the drinking gesture can be useful for monitoring fluid intake and hydration levels [32], and the recognition of bringing the hand to the mouth with a certain grasping shape can be linked to pill intake, useful for medical adherence assessment [33]. Thus, specific attention should be given to gestures, including also other daily living activities that can have similar execution mechanics, in order to test the robustness of the recognition methodologies to potentially confounding patterns. However, it should be noted that the inclusion of additional activities that involve whole-body movement can be considered in order to enlarge the spectrum of detectable activities through the specific sensor configuration chosen for this application. The second point is instead related to the reproducibility of the results [34]. Reproducibility is, in fact, a relevant property that should be observed, especially with calibrated instruments, but is typically overlooked when dealing with sensor data. Finally, users highly value their privacy and hence require a high degree of protection for their personal data, but they also accept to provide complete data transparency in case of perceived risks to their health and potential countermeasures offered by the monitoring technology [35]. Likewise, users exhibit willingness to pay for the implementation of these technologies in living environments if perceived beneficial to improve safety and health.

Originally focused on simple activity detection, HAR has evolved to more challenging tasks of identifying complex and multiple simultaneous activities, a change driven by technological advancements [36]. In this context, multi-modal sensor fusion, which involves the intelligent combination of data from different sensor types, becomes crucial to attain a more comprehensive, accurate, and robust understanding of both performed activities and the environment, and even user intention. The latter point can nowadays be addressed by wearable BCIs. They provide direct access to user intent, enabling control and communication through neural signals, but they offer limited or no awareness of the external environment. In the following sections, the analysis of the current state of adoption of each sensing modality, i.e., RGB-D cameras and BCIs, is presented to lay the foundations for the proposal of their integration, discussing opportunities and open challenges to address.

## 3. HAR Based on RGB-D Cameras

Vision-based HAR still represents one of the major challenges in the field of computer vision [37], and RGB-D cameras remain a common solution to implement AAL systems and applications. Indeed, a large portion of HAR systems, especially for fall detection, is represented by depth cameras [23], such as the commercially available Orbbec [38] (Orbbec, Troy, MI, USA), Intel RealSense [39] (Intel Corp., Santa Clara, CA, USA), and Microsoft Kinect [40,41] (Microsoft Corp., Redmond, WA, USA). RGB-D cameras can actually gather information in a number of modalities and are typically devised to capture vision-based information under infrared (IR) or near-infrared (nIR) channels, skeletal and RGB. However, depth frames provided by RGB-D cameras are more commonly considered suitable for handling changes in room illumination and protecting the privacy of the users.

Table 2 reports advantages and disadvantages of RGB-D cameras. They may operate based on different techniques, i.e., stereo vision, IR speckle pattern projection and Time of Flight (ToF) at IR or nIR. In stereo vision, the depth map of the scene is reconstructed from a couple of RGB images acquired from two different directions by extracting distance information. In IR speckle pattern projection, an IR or nIR dot pattern is projected by the sensor onto the scene, and then the dots are detected by the IR camera. The depth information is extracted by analyzing the changes between the projected and detected dot patterns. In Time of Flight, the object-to-sensor distance is derived by measuring the time taken by a light pulse (emitted by an IR/nIR laser or an LED) to travel from a source onboard the sensor to the object and back to the sensor receiver. The depth-sensing technique implemented influences not only the cost of the corresponding camera but also the performance in terms of range of operation, depth frame resolution, frame rate, and accuracy [42].

Although advances in manufacturing technologies have led to a reduction in the physical dimensions of RGB-D cameras, there are still some implementation challenges to face. In fact, regardless of the specific depth-sensing technique, factors of temperature and ambient light may influence the attainable performance and hinder the effectiveness of the adopted device in uncontrolled conditions, such as outside laboratories and in AAL home scenarios. Such influencing factors lead to noisy or incomplete depth images, which, in their turn, do not enable correct identification of skeleton joints or other body-tracking metrics. As shown in [43], not only noisy conditions but also processing platforms and algorithms may generate noticeable differences among the output results. Another important factor to take into account regards the amount of data generated by RGB-D cameras. Indeed, depth frames, typically acquired at a rate of 15 frames per second (or even more), need adequate data storage capacity and power for processing, especially for real-time applications (e.g., fall detection). Two options are usually considered, namely, local data processing, which requires high storage resources onboard the sensor, or remote data processing, which removes the burden of computation from the sensor but necessitates enough wired or wireless data transfer rate. The latter approach can face implementation issues in situations where a network infrastructure is not already in place, such as in old buildings, or does not meet the application requirements, as highlighted in real-life evaluation studies.

Table 3 provides a comparison among notable commercial RGB-D cameras regarding the operating principle used. Cameras of the RealSense D400 series by Intel (Intel Corp., Santa Clara, CA, USA) are popular for HAR applications in the AAL domain for their versatility and relatively compact size, combined with affordable cost. They use stereo vision to calculate depth, while the L515 uses Light Detection and Ranging (LiDAR) technology for depth sensing, providing high-accuracy depth frames and low power consumption. The Kinect v1 and v2 cameras (Microsoft Corp., Redmond, WA, USA), using structured light and the ToF operating principle, respectively, are nowadays considered legacy, but they are historically significant for having made RGB-D imaging popular and affordable, first in the gaming space, then in the AAL domain. The more recent Kinect Azure camera (Microsoft Corp., Redmond, WA, USA) employing ToF mostly targeted developers and industrial applications, offering higher resolution and more advanced features than the consumer versions. In August 2023, Microsoft discontinued Kinect production, including Azure. Orbbec is nowadays a global leader in the design and manufacturing of 3D cameras, with a diverse offering of products tailored to different applications. The affordable Astra Pro series (Orbbec, Troy, MI, USA), among those more frequently used in AAL and HAR applications, exploits ToF in the nIR range, providing good performance for gesture and action recognition. Other solutions are provided by Asus, with the Xtion Pro device [44] (AsusTeK Computer Inc., Taipei, Taiwan) offered for more than ten years, and Structure IO (Structure, Boulder, CO, USA), both using structured light 3D sensing technology [45], but their usage is mostly limited to research and development activities.

Selecting an appropriate RGB-D camera requires a comprehensive evaluation of several technical and practical specifications to match them with the demands of the intended application. From the technical perspective, for applications demanding precise 3D data (detailed object reconstruction, accurate spatial mapping), higher resolution and superior accuracy are paramount, as they directly impact the fidelity and reliability of the captured depth information. Furthermore, the maximum operating distance of the camera is a critical factor, particularly when the application involves larger environments or requires capturing objects or moving subjects at varying distances. An adequate range ensures comprehensive data acquisition across the desired operational volume. Finally, a wider field of view (FoV) is advantageous for applications capturing larger areas or for the simultaneous observation of multiple subjects within a scene. A broader FoV can reduce the need for multiple camera placements (thus reducing also complexity and costs) or extensive frame stitching in post-processing steps.

Among the practical issues to consider, the physical dimensions and form factor of the camera are crucial for seamless integration into diverse systems and environments and for compact experimental setups. Miniaturization and specific form factors can significantly influence overall system design and acceptability from the subjects being monitored [35]. Alongside this, the financial investment associated with RGB-D cameras varies considerably across different models and manufacturers. Budgetary constraints often play a significant role in the selection process, necessitating a balance between desired performance and economic viability. Lastly, the availability of robust software development kits (SDKs) and comprehensive libraries for depth frame processing is fundamental for efficient system development and application integration: strong software support facilitates data interpretation, algorithm implementation, and overall system functionality.

Beyond HAR, RGB-D cameras are being adopted across a multitude of sectors within consumer electronics, robotics, machine vision, and the automotive industry. The increasing number of applications using RGB-D cameras supports projections of market expansion at a compound annual growth rate (CAGR) of over 10% until 2031 [46] and the expectation that these devices will be available at a more affordable price and with improved performance in the future. This is a relevant aspect to consider for the viability and sustainability of HAR-based AAL applications and systems in the long term.

### 3.1. ML Techniques

RGB-D cameras capture both traditional color (RGB) images and depth (D) information for each pixel, providing rich datasets that significantly improve ML and deep learning (DL) approaches for HAR. Consequently, RGB-D-based HAR methods can directly exploit acquired images (RGB and/or D ones) or skeletal information obtained from them. This combination of visual appearance and 3D geometric data allows algorithms to perceive and understand human activities with greater accuracy and robustness. For example, in [47], a recurrent neural network (RNN) is proposed for recognizing human activities from depth camera images. The learning model, however, is not fed directly by images acquired at each frame; instead, skeletal joint positions are extracted from each image, and then joint angle variations are computed at each recorded frame in order to avoid dependencies from specific positions of the joints. The full body kinematics is taken into account. For encoding time sequential information, an RNN with long-short-term-memory (LSTM) is chosen, where the number of LSTM matches the length of the activity video frame. A total of 12 activities were considered for testing the proposed architecture: lift arms, duck, push right, goggles, wind it up, shoot, bow, throw, had enough, change weapon, beat both, and kick. The proposed methodology was compared with other state-of-the-art classification schemes, i.e., hidden Markov model (HMM) and deep belief networks (DBN). RNN outperformed both HMM and DBN, with an average accuracy above 99%, whereas the DBN offered about 97% in correctly classifying the 12 different activities.

In general, a major role in RGB-D-based HAR is played by artificial intelligence architectures employed to process images in order to extract human silhouettes, identify skeletons, or compute features. Their fast progress in the last few years depends also upon the growing availability of large amounts of data and large-scale datasets required for training, like those reported in Table 4 and discussed later in this section. In this view, convolutional neural networks (CNN) represent the state-of-the-art for image processing and computer vision-based applications [48]. CNNs also allow learning hierarchical characteristics from unprocessed data, avoiding the handcrafted feature extraction procedure commonly employed when more traditional machine learning approaches are adopted for pattern recognition purposes. Some studies also used multi-dimensional CNN to further exploit the potential of such algorithms for image processing. The use of 2D CNN allows us to enhance the spatial information that can be retrieved from images, but in some cases they showed good performances only in poor, challenging scenarios [49]. For addressing this issue, 3DCNN found large employment, since they are able to learn spatial and temporal information by convolution within and across the video frames [50]. However, 3DCNNs become highly computationally demanding when long-range temporal dependencies across the frames have to be captured, imposing non-negligible problems when used for activities occurring for extended periods of time [51]. More recently, hybrid methods have been proposed to leverage the strengths of different models and improve the learning of discriminative features from the video frames. Spatial features can be extracted by pretrained nets and then concatenated for feeding LSTM as a sequence [52]. Multi-stream procedures were also proposed for learning spatial and temporal features for recognition of human activities [53,54].

However, due to the high number of environmental characteristics and challenging scenarios that can be encountered, e.g., texture variations, low resolution, scaling, and temporal flow, the recent literature in the field of HAR from RGB-D camera images focused its efforts on the development of novel architectures aimed at improving recognition performances in a variety of different contexts, pushing toward the generalization of such architectures. Hussain et al. [55] presented a two-module architecture. The former was devoted to feature extraction and based on a dynamic attention fusion unit. The latter was made by a temporal-spatial fusion network that encoded complex patterns and learned the most discriminative features for the final HAR in challenging, real-world conditions. Indeed, HAR from images requires understanding the interactions between the human body and the environment over time. Both spatial and temporal features are important because activities to recognize involve sequences of motion over space and time. The proposed methodology was evaluated on four available datasets, with various classes of human activities, that resemble real-world scenarios in terms of motion, appearance, viewpoint, and lighting conditions. Classification accuracy was well above 98% for three out of the four tested datasets, whereas for the last one about 80% accuracy was achieved. Although a wide range of different activities were taken into account, this study highlights that the input video data affect crucially the performance of the recognition model. Indeed, differences in image brightness and background between training and inference data are a well-known problem, referred to as distributional shift. It leads to a performance degradation, also when considering that an action is dependent on the performer and the same activity made by two subjects can be significantly different from each other. Thus, subject-specificity of movement data often lowers the capability of the model in correctly classifying actions [56].

One of the most promising solutions proposed to deal with this issue is domain adaptation, consisting of the training of a domain-invariant learning model, using domain labels as a clue [57]. A phase randomization approach was developed in [56], suitable for working on skeleton data extracted from RGB-D images. In this approach, a novel data augmentation procedure was developed to highlight subject features by decomposing motion data into amplitude and phase components. The phase randomization works by randomizing the phase of the frequency components, keeping untouched the relative amplitude, which most likely includes the individuality of the subjects. A more general framework was proposed in [58], with a two-stage pipeline made of a skeleton sequence generation followed by a stacked ensemble classification of human activities. The 2D skeletal key points are extracted from image frames by using MoveNet [59], and then converted into 3D by a Gaussian RBF kernel. Then, spatial and temporal features, reflecting specific motion dynamics, are computed on 3D reconstructed skeleton data and used for the eventual classification of human activities. The latter includes a convolutional LSTM block, a spatial bidirectional gated temporal graph convolutional network, and a convolutional eXtreme gradient boosting. This general framework was tested on several datasets encompassing images recorded by different devices, namely the NTU-RGB+D60 dataset [60], where Kinect v2 was employed and 60 activities are included; the NTU-RGB+D120 dataset [61], where RGB-D cameras are used; the Kinetics-700-2020 dataset [62], which includes video clips taken from the YouTube website for over 700 activity classes; and the MA-52 [63], which includes 52 micro-actions (see also Table 4 for details). The framework provided above 95% accuracy for each dataset, proving to be reliable in several different working conditions and with different sources of information.

Interaction between humans and objects represents an additional field where image processing has been employed. Li et al. [64] introduced a novel paradigm through a model that leverages natural language processing for supervising visual feature learning, enhancing their expression capability. The proposed methodology was tested on two human-object interaction datasets, namely the CAD-120 dataset [65] and the Something-Else dataset [66]. The former contains 120 RGB-D videos for 10 complex activities, with labeled sub-activities, whereas the latter includes more than 10^5^ videos for 174 interaction activities. Results showed that the inclusion of natural language supervision in the learning process improves the recognition performances on both datasets but is limited to indoor settings and interaction scenarios involving only a single human.

**Figure 2:**
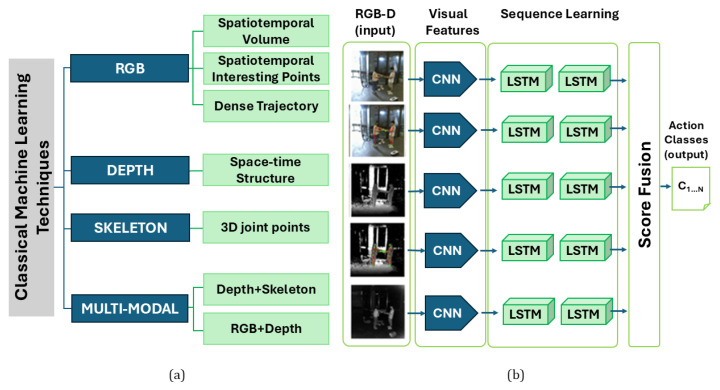
Exemplary graphical representation of (**a**) machine learning- and (**b**) deep learning-based methods, for HAR using RGB-D cameras, from [23].

Rather than focusing exclusively on learning architecture and modules, some works also emphasize the role of feature extraction from RGB-D images. For instance, Elnady and Abdelmunim [67] combined an LSTM network with You Only Look Once (YOLO) for activity recognition in video sequences. In addition, a tracking model was included for maintaining temporal consistency of the detected objects across the video sequence. The proposed methodology was tested on four publicly available HAR datasets: the UFC101 dataset [68], the KTH dataset [69], the WEIZMANN dataset [70], and the IXMAS dataset [71]. The inclusion of YOLO for feature extraction dramatically enhanced the accuracy of object recognition within complex environments, paving also the way for a possible real-time usage of the method. Leveraging skeleton data represents an attractive way for dealing with HAR from images, since skeleton data are computationally efficient and more robust with respect to some environmental characteristics, such as changes in illumination, background noise, and camera view. For instance, Chen et al. [72] proposed a spatio-temporal graph CNN specifically designed for improving activity recognition from skeleton data by taking into account indirect connections between skeletal key points. The proposed network demonstrated the capability of extracting spatio-temporal features on a local and global scale by a graph CNN and a spatio-temporal Transformer. These two distinct streams allowed us to extract topological and motion-related structures, together with relationships between different skeleton joints, thus leveraging two different representations of the same object. Classification architecture was tested on the NTU-RGB+D60 dataset [60], the NTU-RGB+D120 dataset [61], and the Kinetic-Skeleton dataset, where skeleton representation was derived from videos of the Kinetics 400 dataset by using the OpenPose algorithm [73]. Outcomes showed that the proposed architecture was able to improve state-of-the-art solutions when tested on the same datasets. A short summary of the most used HAR datasets publicly available is reported in Table 4, with details on the type of collected data and activities included. Figure 2 provides an exemplary (though not exhaustive) graphical representation of machine learning- and deep learning-based methods for HAR using RGB-D cameras.

**Table 4:** Publicly available datasets used for testing computational and learning frameworks for HAR from video recordings.

Dataset	Data	Activities
MSRC-12 [47]	6244 video samples with skeletal joints position	12 activities from 30 subjects
NTU-RGB+ D60 [60]	56,880 video samples, including RGB, infrared, depth, and skeleton data	60 ADLs
NTU-RGB+ D120 [61]	Expanded version of NTU-RGB+ D60, with 114,480 video samples	120 ADLs
MA-52 [63]	22,422 video samples	52 micro-actions from 205 subjects
CAD-120 [65]	120 video samples	10 high-level activities, 10 sub-activities, and 12 object affordance from 4 subjects
PKU-MMD [74]	1076 video sequences, including RGB, depth, infrared, and skeleton data	51 ADLs by 66 subjects
Berkeley-MHAD [75]	660 action sequences recorded from 4 cameras, 2 Kinect cameras, 6 accelerometers, and 4 microphones	11 ADLs by 12 subjects with 5 repetitions for each ADL
HWU-USP [76]	Recordings from ambient sensors, inertial units, and RGB videos for a total of 144 recording sessions	9 ADLs by 16 subjects
Northwestern-UCLA Multiview [77]	RBG, depth, and skeleton data recorded by 3 Kinect	10 ADLs by 10 subjects
UTD-MHAD [78]	RGB, depth, skeleton data recorded from 1 Kinect camera, and inertial data from a single inertial sensor	27 ADLs by 8 subjects, with 4 repetitions for each ADL
Toyota Smarthome [79]	16,115 video samples, including RGB, depth, and skeleton data recorded from 7 Kinect cameras	31 ADLs from 18 subjects
UFC101 [68]	13,320 video samples	101 ADLs
KTH [69]	2391 video samples from 25 people	6 ADLs in four different contexts
WEIZMANN [70]	90 low resolution video sequences	10 ADLs by 9 subjects
IXMAS [71]	2880 RGB video sequences	15 ADLs by 5 subjects, with each ADL repeated 3 times
Kinetics-700-2020 [62]	Collection of a series of datasets containing up to 700 video samples for each ADL	Up to 700 ADLs, depending on the considered specific datasets
Kinetic-Skeleteon [80]	Derived from Kinetics dataset, using OpenPose to extract skeleton key points, and made by 300,000 videos	400 ADLs
HMDB51 [81]	6776 annotated video samples from various sources as movies and YouTube	51 ADLs
STH-STH V1 [82]	108,499 video samples	174 ADLs from 1133 crowdsource workers
UCF50 [83]	6681 video samples	50 ADLs
YouTube Action [84]	1600 video samples from YouTube	11 ADLs
MSR-3D [85]	320 video frames, including skeleton data, RGB, and depth images	16 ADLs
JHMDB [86]	928 RGB video, for a total of 31,838 frames	21 ADLs

In general, it is fair to say that skeleton data and RGB modality represent uni-modal approaches (Table 5) based on two different strategies for achieving reliable results in the field of computer vision-based HAR [87]. However, some limitations have been outlined throughout the years. Only to mention a few, the absence of a 3D structure as input is recognized as one of the major limitations of HAR from RGB-D cameras, together with the absence of environmental features [88]. On the other hand, multi-modal approaches, even though less investigated, proved to be particularly suitable for RGB-D-based HAR. In essence, multi-modal methods rely on data fusion on multiple levels, i.e., on a data, feature, or decision-level fusion (Table 6). The problem of multi-modal fusion was tackled in [88] by developing a multi-modal network integrating RGB images and skeletons relying on a model-based approach, where a graph CNN was used for learning weights to be transferred to another network devoted to RGB processing. Also in this case, extensive testing was performed on five datasets: the NTU RGB+D60 dataset [60], the NTU RGB+D120 dataset [61], the PKU-MMD dataset [74], the Northwestern-UCLA Multiview dataset [77], and the Toyota Smarthome dataset [79]. A similar multi-modal approach was proposed in [89], where images of skeletons, motion history (MH), and depth motion maps (DMM) are firstly retrieved from RGB-D cameras. Then, a 5-stack CNN was trained separately on MH and DMM only, whereas for skeleton images the output of the CNN was used for feeding a BiLSTM. Then, an additional fusion step was applied at the level of the score values of the three previous networks, and the final decision is based on the fusion value. The framework was tested on the UTD-MHAD dataset, which contains 27 human activities collected by using a depth camera, with over 96% accuracy on the validation set. Multi-modal HAR was leveraged also by Batool et al. [90], who introduced a HAR solution based on RGB, RGB-D, and inertial data. Silhouette extraction was performed from RGB-D images by applying Laplacian fitting [91], whereas inertial data underwent a Kaiser windowed filter [92]. Then, specific features were extracted from RGB-D, namely dynamic likelihood random field and angle along the sagittal plane, and from inertial data, i.e., lag regression [93] and gammatone cepstral coefficients [94]. Features were then selected and fused by a genetic algorithm and given as input to a CNN-gated recurrent unit that leveraged Kalman gain instead of a rectified linear unit layer. Results gathered from testing on the UTD-MHAD dataset [78], HWU-USP dataset [76], Berkeley-MHAD dataset [75], NTU-RGB+D60 dataset [60], and NTU-RGB+D120 dataset [61] showed that the proposed methodology outperformed existing similar solutions by achieving an accuracy not below 95% for each of the five considered datasets. A multi-modal approach was also recently proposed by Liu et al. [95], who presented a semantic-assisted multi-modal network specifically designed for overcoming inherent limitations of skeleton and RGB uni-modal approaches. The method works by leveraging adaptive learning among three modalities, where text was added to skeleton and RGB video, thus including detailed textual descriptions of the activities, enriching the category expression. Also in this case, the method was tested on several different datasets, i.e., the NTU-RGB+ D60 dataset [60], the PKU-MMD dataset [74], and the Northwestern-UCLA Multiview dataset [77], showing promising improvements with respect to other existing solutions. Song et al. [96] presented a modality compensation network to properly leverage the complementary information given by different activity representation modalities. In particular, the RGB flow was designed as the source modality, whose feature extraction was improved by including information from the auxiliary modality, i.e., the skeleton data. A two-stream architecture was composed of a CNN and an LSTM, where the former was used for encoding the spatial features, while the latter collects and integrates the information flow over time. Then, the final decision is obtained from the score fusion of each network block. Notably, skeleton data were needed only for training. The architecture was tested on the NTU-RGB+D60, MSR-3D, UCF101, and JHMDB datasets (Table 4), showing improvements in HAR for all the tested datasets. Skeleton and RGB-D modalities have been leveraged also in [97], where a multi-modal framework, enhanced by temporal cues, was proposed. The weighted skeleton joints, learned from a dedicated graph convolutional network (GCN), are used for improving the identification of the spatio-temporal region of interest (ROI) from the RGB video modality. In addition, a temporal cues enhancement module was introduced for the RGB modality, made by a two-stream architecture with a CNN for the spatial domain and an RNN for the temporal cues. The proposed architecture was tested on three publicly available datasets, namely NTU-RGB+D60, PKU-MMD, and the Northwestern UCLA dataset (Table 4). Outcomes showed that the proposed methodology outperformed several previously proposed methods in both cross-subject and cross-view modalities.

### 3.2. Uni-Modal vs. Multi-Modal Sensing

In light of the previous overview, it is fair to say that both uni-modal (Table 5) and multi-modal (Table 6) approaches present some challenges and issues that still have to be addressed. For what concerns RGB-based HAR, the background in videos can significantly influence action recognition, often neglecting the actual modeling of the actions themselves. To address this issue, attention mechanisms working on foreground motion have been proposed in order to focus on the human appearance and moving regions, minimizing at the same time the impact of the background objects [98]. However, focusing on moving objects only can lead to wrong conclusions when dealing with complex environmental contexts, highlighting the need for a tailored recognition of those objects that are truly relevant for HAR, which, however, represents itself as a complex and challenging task to be accomplished. Another critical aspect of RGB-based HAR is related to the computational costs for image processing. Although in the last few years computing power underwent remarkable improvements, enabling the widespread application of deep learning across various domains, the increased accuracy of this kind of architecture is achieved at the cost of even higher computational complexity. The latter aspect currently prevents the widespread application of RGB-based HAR technology on mobile devices or systems with limited processing capabilities. However, some solutions have been proposed, such as reducing redundant video information for achieving a more lightweight recognition or optimizing feature extraction pipelines [99,100].

The previous challenges can be mitigated by shifting the recognition paradigm toward the use of skeleton data, since the latter are more robust with respect to changes in the appearance and, in general, require fewer computational resources by neglecting background and object information [87]. When using skeleton data only, a key aspect is the modeling of the changes in the dynamics of actions [56,72]. However, it has also been highlighted that appearance information can enhance recognition of activities with similar motion dynamics [101], thus leading to a significant amount of work where an RGB-skeleton multi-modal approach was used, in order to balance computational needs and identification performances, by fusing temporal movement and appearance features (Table 6). Therefore, environmental characteristics appear to be a critical type of information for HAR applications, capable of enhancing the final recognition rate. In addition, skeleton-based action recognition also neglects information related to the objects involved in some particular actions, which instead represents an important point for many applications. Although some attempts have been made to include object-related information in a uni-modal, skeleton-based HAR framework, as highlighted, for instance, in [102], where objects were treated as an additional joint, combining skeleton data and object information still remains an open research aspect in this field. However, successfully accomplishing this task would offer significant advantages, since it would allow us to avoid privacy-related issues and rely only to a minor extent on the background characteristics, thus keeping computational costs at a reasonable level.

Even though a multi-modal approach thus offers, in general, a more robust approach for HAR from images, there are still some challenges that arise in particular when dealing with real usage scenarios outside controlled environments used for framework development. One of the major issues is to ensure reliable activity recognition when multiple persons are present in the same scenario and perform different activities, representing a condition that cannot be a priori avoided in real-world contexts. For addressing this issue, several works proposed solutions mainly based on the usage of multiple cameras or on refined feature weighting procedures [103,104]. However, unlocking the full potential of action recognition technology for multi-person and multi-action tasks remains a significant hurdle for real-life applications in this field. Connected to the multi-person, multi-action issue, there is also the problem of occlusion, which is unavoidable in densely populated scenes. To address this issue, widely adopted solutions are the fusion of multiple cameras’ information and the a posteriori reconstruction [105,106]. However, while these approaches have made significant progress in mitigating occlusion-related issues, challenges such as large-scale occlusion and the inability to capture multi-angle video data remain still the object of much research effort.

Finally, it is worth mentioning that an additional challenge when deep learning approaches are employed for HAR is to have enough data to achieve a robust training of the learning models. Although throughout the years many datasets have been recorded and made publicly available (Table 4), their dimension is often limited with respect to the adopted architectures, and few of them were collected in real-life scenarios. Further, also the lack of labels represents an additional challenge. To address these issues, data augmentation schemes offer a viable solution [107], together with the usage of fine-tuning strategies of pre-trained models and unsupervised learning architectures [108].

Overall, multi-modal approaches seem to be a convenient solution for addressing some problems arising with uni-modal strategies. However, as outlined above, also integrating RGB and skeleton modalities within a more comprehensive approach encompasses some drawbacks that are still far from being completely solved. Therefore, the latter studies highlighted the value of fusing different types of information to enhance HAR from images recorded by RGB-D cameras. This supports the opportunity of investigating the inclusion of additional devices and data flow when dealing with the identification of human activities and gestures from images and video recordings. In this view, BCI could represent a viable solution, since it provides a completely different kind of information with respect to RGB and/or skeleton data, with the potential of limiting some of the problems given by a fully image-based scheme. At the same time, BCI provides information that is specific for the single user, with the possibility of designing more subject-centered strategies for HAR also within real-life scenarios.

## 4. HAR Based on BCIs

In AAL environments, wearable devices have been increasingly utilized to improve the quality of life of frail and elderly individuals in their living environments and to promote autonomy and safety. This innovative strategy enables residents to seamlessly control their living spaces, making everyday tasks easier through intuitive interactions with various devices and robots. Wearable technologies also allow for collect real-time data on user health status and physical environment, allowing personalized adjustments to improve comfort, safety, and efficiency. This integration fosters a symbiotic relationship between the user and the environment, moving away from traditional interaction and user interfaces, where human feedback and sensor data continuously inform and optimize each other responses, ensuring a supportive and adaptable living space that caters to the unique needs of its inhabitants in a more human-centric approach. Users can command and communicate with their surroundings in a more natural and effortless manner, using the capabilities of wearable technology, such as smartwatches, fitness bracelets, and even more advanced tools such as BCI.

Among the wearable technologies being deployed for user feedback in AAL environments, BCIs are particularly significant and promising, especially for people with severe impairments. BCIs have a wide range of applications, prominently in the field of assistive technologies. They have been used to restore communication to control prosthetic limbs or robotic arms, offering new possibilities for people with different frailties [109]. Another important application area is the rehabilitation of stroke patients, where BCIs can facilitate motor recovery by enhancing neuroplasticity. This is achieved through the use of BCIs to control virtual reality environments or robotic devices that provide patients with feedback and assistance in performing motor tasks [110]. Wearable BCIs, based on the acquisition of electroencephalography (EEG) signals from the user scalp, enable the direct interpretation of the user neural signals, which can be encoded into machine-readable commands, for a natural and personalized control of the devices located in the living setting. With this scope, BCI applications can support users as interfaces to monitor and record data from cognitive signals during interaction tasks or as control interfaces for human–robot interaction or cooperation activities. In fact, direct control over home devices and robots is thus facilitated, enabling users to manage their surroundings with simple gestures or voice commands.

BCIs offer a revolutionary means to control both the living environment and various devices within it, including essential services and personal robots that assist with daily activities [111]. Their technology enables direct brain-to-machine communication, bypassing traditional physical interaction modalities and offering a more inclusive and accessible way for users to interact with their surroundings. BCIs operate on the principle of detecting, decoding, and translating brain activity into external actions. The brain activity can be recorded using various methods, including non-invasive techniques such as electroencephalography (EEG) and more invasive approaches such as electrocorticography or intracortical recordings. EEG, due to its ease of use, safety, and cost-effectiveness, is the most commonly used method in BCI systems [112]. With respect to this approach, the most used BCIs in the scientific research are the non-invasive ones that detect brain activity externally. In Table 7, some of the commercial BCI prototypes and products used in different applications and with various scopes in scientific studies are reported. As reported before, in the literature invasive approaches and, in particular, invasive BCIs that require surgical implantation are also presented. In particular, authors report some of the commercial products/companies as Neuralink, Synchron (Stentrode), Blackrock Neurotech, Paradromics and Precision Neuroscience, which are characterized by high-density brain implants, minimally invasive via blood vessels, and invasive brain interfaces. These high-density implantable BCI systems are developed with the aim of supporting neural decoding and rehabilitation approaches, particularly for paralyzed patients and people with severe disabilities and neurological conditions, to restore and improve motor control and communication.

The integration of BCIs into AAL environments represents a leap forward in human-environment interaction, allowing for a level of autonomy and engagement previously unattainable for many users. With BCIs, individuals can control aspects of their environment and interact with service robots [113] through only thought patterns. This capability is particularly transformative for those who face mobility or communication challenges, as it provides them with a new avenue to interact with their environment effectively and independently.

From this point of view, BCIs represent a cutting-edge technological paradigm that facilitates direct communication pathways between the brain and external devices, bypassing conventional neuromuscular routes. This technology harnesses neural signals, interprets them through computational algorithms, and translates these signals into actionable commands for various applications, ranging from medical rehabilitative tools to augmentative communication devices and control systems for the environment or robotics. The decoding process involves sophisticated signal processing and machine learning algorithms [114] to interpret complex patterns of brain activity. These algorithms are designed to identify specific signal features associated with intentions or thought processes and translate them into commands [115]. Furthermore, the application of BCIs in these settings shifts towards more naturalistic and human-centric user interfaces, moving from the human-computer interface to the human-environment interaction, where the focus is on creating smart environments that adapt to and understand the needs of their users through feedback mechanisms. In this context, Streitz proposed a transition from human–computer interaction to human-environment interaction, highlighting the future, where individuals interact with smart environments composed of various devices. These environments often feature integrated computing devices in everyday objects, making them less noticeable, which are referred to as disappearing computers [116]. This integration poses new challenges in providing suitable interaction possibilities, and it results in crucial importance to focus on supporting human interactions within these settings, considering the importance of user control, transparency, ethics, privacy, and other significant issues. In the realm of human-environment interaction, a key development is the need to effectively use human feedback to adjust environmental behavior. This feedback is crucial in cooperative tasks to manage and mitigate factors that can hinder performance, as highlighted in several studies. Human feedback is particularly vital to improve safety in various situations. Using BCIs, these environments can become more responsive and tailored to individual preferences and requirements, enhancing not only the usability but also the safety and well-being of their inhabitants.

BCIs, therefore, not only enrich the user control over their living space but also contribute to the ongoing dialog on the ethical, privacy, and transparency concerns inherent in these technologies. As BCIs become more integrated into AAL environments, it is essential to address these issues to ensure that the technology empowers users without compromising their rights or autonomy. The potential of BCIs in AAL environments underscores the importance of developing smart living spaces that prioritize user interaction and feedback, thereby fostering a more inclusive, safe, and responsive living environment for all.

Despite promising applications, BCIs face several challenges. The variability in individual brain signals, the need for personalized calibration, and the limited accuracy and reliability of signal decoding are significant hurdles, as reported also in Table 8, where advantages and disadvantages of the BCI applications are reported. Moreover, non-invasive BCIs often suffer from low signal resolution due to the interference of signals by the skull and scalp [117].

Future research directions include improving the usability and robustness of BCI systems, enhancing signal processing algorithms for better accuracy, and developing adaptive systems that can learn and adjust to change user brain patterns over time. Moreover, integrating BCIs with other technologies, such as augmented reality, virtual reality and mixed reality, opens new avenues for immersive and interactive applications [118]. In this context, in [119] a study related to an innovative methodology to train an artificial neural network is proposed to identify and tag target visual objects in a given database. In particular, with the aim of improving the tag data phase, the study uses the advantages of human cognition and machine learning by combining BCI, human-in-the-loop, and deep learning. The users are equipped with a BCI system and images are shown using a rapid serial visual presentation. Some images are target objects, while others are not. Based on the activity of the brain waves of the users, when the target objects are shown, the computer learns to identify and tag the target objects already in the learning stage.

The authors report, in this scenario, the experience of a pivotal work to realize a human-in-the-loop approach, where the human can provide feedback to a specific robot, namely, a smart wheelchair, to augment its artificial sensory set, extend and improve its capabilities to detect and avoid obstacles. In the implemented approach, input from humans is facilitated through a BCI. In pursuit of this goal, the study also encompasses the development of a protocol to trigger and capture event-related potentials in the human brain. The entire framework is preliminary and will be tested in simulated robotic environment, using electroencephalography signals to collect from different users [120].

The focus of the research is on a smart wheelchair capable of indoor navigation and autonomously avoiding obstacles using its in-built sensors and intelligence. Human feedback comes into play when the operator detects an obstacle not recognized by the sensory capabilities wheelchair. Upon receiving human input, the robot navigation system adjusts to incorporate this feedback, creating a virtual obstacle. This adjustment alters the robot local path planning to circumvent the detected obstacle. BCI is integrated as a feedback method, together with a protocol designed to invoke the event-related potential (ERP) signal when encountering obstacles. Initially, the efficacy of the system will be tested using keyboard inputs to provide feedback, establishing a performance baseline. Subsequently, a BCI classifier will be used, showcasing the system capabilities with this advanced feedback mechanism.

### Human-In-The-Loop Approaches

Human-in-the-loop is an increasingly researched area that refers to the integration of human feedback into computation processes [119]. The human-in-the-loop approach can be applied to different scenarios in which human control can be involved in the designed task. To provide human feedback, it is well-established that brain activity and mental states are decoded to varying degrees of accuracy, depending on the paradigm, equipment, and experimental setup used [121]. The BCI should learn from the data and be tailored to a specific subject or session as more data becomes available.

Nowadays, different studies investigate how to implement the human-in-the-loop control as feedback for robots in different activities to support people with special needs. In the literature, the study proposed in [122] investigates a BCI system that allows interaction with the external environment by naturally bypassing the musculoskeletal system. The approach performed a hybrid EEG-based BCI training involving healthy volunteers enrolled in a reach-and-grasp action operated by a robotic arm. The results obtained showed that hand grasping motor imagery timing significantly affects the evolution of BCI accuracy, as well as the spatio-temporal brain dynamics.

Often, in different approaches, the EEG signals acquired by BCIs are evoked through visual stimuli. The work [123] proposes a close-loop BCI with contextual visual feedback through an augmented reality headset. In such a BCI, the EEG patterns from multiple voluntary eye blinks are considered as input, and the online detection algorithm is proposed, whose average accuracy can reach a high value. In this context, the objective of the study [124] is to explore the feasibility of decoding the EEG signature of visual recognition under experimental conditions, promoting the natural ocular behavior when interacting with a dynamic environment. The results show that visual recognition of sudden events can be decoded during active driving. Therefore, this study lays a foundation for assistive systems based on driver brain signals. In the article [125], a novel strategy was proposed that uses the human mind to choose the mode of ambulation of prosthetic legs. The article described methods for acquiring information about human brain activity and recognizing intentions. In order to realize stable and flexible walking of prosthetic legs in different terrains according to human intention, a brain–computer interface based on motor imagery (MI) is developed.

**Figure 3:**
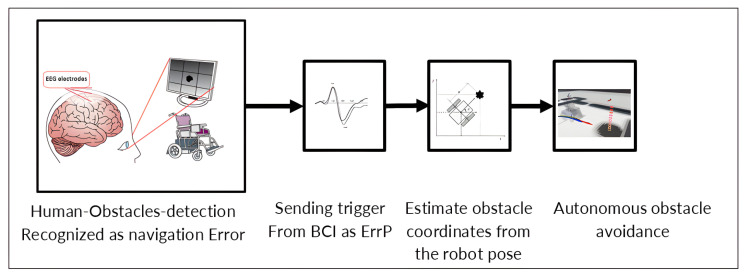
Passive BCI: Utilizing error-related potentials (ErrP) for error detection in the robot navigation path [126]. In the first square participants watched the robot navigate through a simulated environment and instinctively responded to any navigational errors the robot makes. The generated errp is recorded by BCI and sent as trigger in order to change the path planning. In the last two squares the new obstacles coordinates are estimated by the algorithm and the obstacle avoidance is implemented in the simulated environment.

Furthermore, [120] reports an approach with the goal of involving the user directly in the robot navigation process by using human feedback to improve the built-in robot sensory system, thus improving its ability to identify obstacles. For user feedback, it is crucial to accurately estimate the location of obstacles that the robot might not detect. This feedback is then used to generate a virtual obstacle within the virtual environment of the robot, based on its sensory data. Consequently, the robot path planning system, unable to differentiate between actual and virtual obstacles, adjusts its course to avoid the obstacle. This strategy remains valid as long as the virtual obstacle placement accurately reflects the real location of the obstacle (Figure 3).

The involvement of human control and the implementation of feedback mechanism can be facilitated through the BCI system. In particular, it is important to design a protocol for EEG signal acquisition that can provide the useful trigger for the robotic system and for the effectiveness of the implemented approach. Focusing on control navigation, the EEG signals chosen to evoke as a trigger were the error-related potentials (ErrPs). The captured EEG signals revealed slow brain waves indicative of cognitive responses to perceived navigation risks, useful as navigation triggers. The test case was designed through evoked visual stimuli, leaving the user observing the screen displaying a smart wheelchair navigating around different holes in a virtual laboratory environment. Human feedback was generated using standard input devices, including keyboards and touchscreens. The task required the user to signal (via keyboard press) when a hole was noticed. Then, to respond to the same task, equipped with the BCI system, whenever they perceive the wheelchair crossing or evading a hole, they have to press the keyboard [113]. Repetition of the task with the BCI tests the viability of incorporating human feedback via the BCI into the navigation system, training the BCI classifier to recognize ERPs as navigation triggers. EEG signals were processed in MATLAB R2023b, with detected triggers sent to a Robot Operating System (ROS) node for publishing a Boolean trigger value. Within ROS, another node will receive the trigger and adjust the robot navigation strategy to approach and avoid obstacles, ensuring effective avoidance as long as there is proximity between the actual and virtual obstacles.

## 5. RGB-D and BCI Integration: Opportunities, Challenges, and Open Issues

In the previous sections, the current state of adoption of RGB-D camera sensing and non-invasive BCIs as single technologies in research dedicated to HAR in daily life environments has been examined. The presented analysis highlights that the two modalities offer complementary perspectives. RGB-D cameras capture the external context of the user, the physical environment, body movements and interactions, while non-invasive BCIs capture, through neural signals, the internal context as well as cognitive state, intentions, volitional commands or mental engagement of users during activities [20,127]. Neither RGB-D cameras nor non-invasive BCIs, though, when used alone, can ensure a comprehensive and reliable solution for the complex and dynamic needs of AAL. As highlighted by Diraco et al. in [128], the fusion of different sensing modalities enables the design of more comprehensive and robust HAR systems in smart living environments, with increased accuracy and reliability. This is already demonstrated in the literature for the case of inertial, RGB, and skeleton data, or for the case of RGB and radar sensor data: HAR performance may be dramatically improved by resorting to the integration of different sensing technologies, despite the challenges in developing effective models to capture spatio-temporal dependencies in complex settings. Based on these premises, the fusion of RGB-D camera sensing and wearable, non-invasive BCIs is envisioned here to obtain a dual perspective on user activity in AAL.

### 5.1. RGB-D and BCI Fusion Pipeline

A few attempts to fuse the information generated by integrated RGB-D and BCI sensing technologies. Pereira et al. [129] proposed an integrated framework in which data from an RGB-D camera is exploited to improve the BCI-enabled control (by a P300 speller [130]) of a smart wheelchair during navigation in indoor environments. The visual information provided as a background to the P300 graphical control interface helps the user to control the wheelchair with better precision and accuracy than using the BCI alone. In [131], Mezzina et al. proposed a smart sensor system aiming to realize a human-robot interface (HRI) for AAL. The acquired brain signals were wirelessly transmitted to a PEPPER personal care robot (SoftBank Robotics Group, Tokyo, Japan), which navigated the environment, showing the user the available goods and services through an onboard camera. Supported by video information, the user could better formalize unambiguous requests to the robot. Experiments showed that commands could be sent to the actuator in less than 900 ms and executed with an accuracy higher than 80%. A similar approach was presented in [132], again exploiting multi-modal sensing to improve the ability of the BCI user in controlling and refining a NAO robot (SoftBank Robotics Group, Tokyo, Japan) operation. In [133], the integration of a BCI and Kinect sensor to record motion capture information was used for designing an effective rehabilitation system based on serious games. The movements of the hands or the body were sensed by the depth camera for a more natural and dynamic interaction of the user with the serious game; while these works show the feasibility of the integration of RGB-D and BCI technologies with near real-time performance, the degree of multimodal fusion potentially achieved is far from being fully achieved, as this perspective paper aims to suggest.

**Figure 4:**
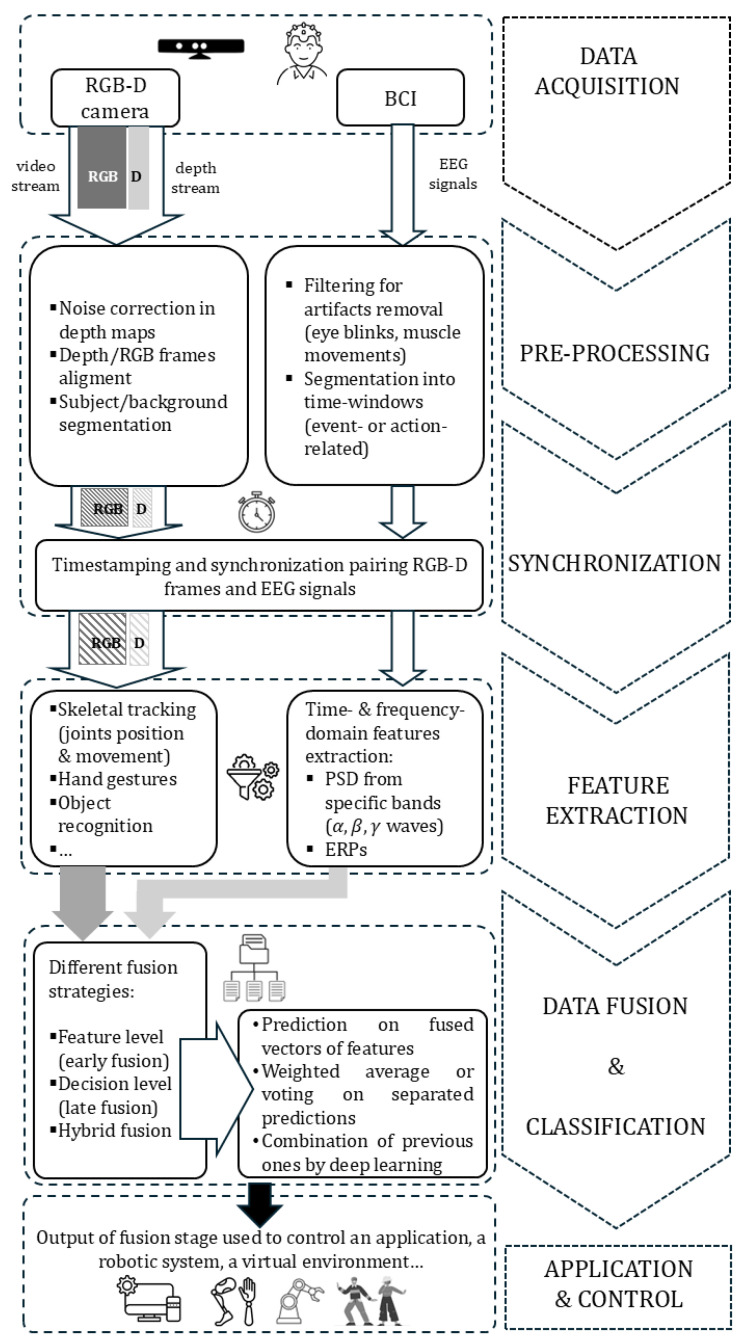
Pipeline of RGB-D and BCI integration steps.

A multi-modal approach can leverage the complementary nature of the data types generated by the two technologies: RGB-D provides rich environmental and physical information (visual and depth), while BCI provides direct user intent and cognitive state. To achieve complete integration based on fusion, as shown in Figure 4, a pipeline composed of several key steps needs to be developed, including data acquisition, preprocessing and synchronization, feature extraction, data fusion and classification, and finally application and control. These same steps basically rule out the multimodal fusion of RGB, skeleton and inertial data, or RGB and radar sensor data, that are commonly found in the literature to maximize HAR performance, providing increased accuracy with respect to unimodal-based solutions and almost real-time recognition capabilities [134,135].

The process starts with data acquisition, i.e., collecting raw data from both sensing modalities simultaneously. The video streams and depth maps captured by the RGB-D camera provide information on physical actions and gestures of users, and the 3D structure of the environment. Raw EEG data, typically generated by non-invasive BCIs, reflect the user cognitive state or intention, which is the key information the BCI system needs to interpret. Raw data from both sources needs to be cleaned and aligned for effective fusion, thus requiring a pre-processing and synchronization step. Following the removal of noise, artifacts, and mismatches from the raw data streams with data-specific techniques as presented in [136] for BCIs, since the two sensors operate at different sampling rates and capture different aspects of the user and environment, their data streams must be precisely time-stamped and synchronized. This ensures that a given BCI signal is correctly paired with the corresponding RGB-D frame. After pre-processing, meaningful features are extracted from each data stream, which enables the core step of the pipeline: data streams are combined to create a more robust and accurate output by different possible fusion strategies at the feature or decision level, or by a hybrid approach [29]. Early fusion approaches working at the feature level are based on concatenating the extracted features from both the RGB-D and BCI streams into a single, combined feature vector. Then, a classifier is trained on the fused vector to make a prediction. Late fusion approaches apply fusion at the decision level. Each data stream is processed independently: an RGB-D classifier makes a prediction based on visual features, a BCI classifier makes a separate prediction based on brain signals, and then the final decision is made by a fusion algorithm combining the outputs of the individual classifiers. Other approaches, namely hybrid ones, exploit deep models that learn to fuse features at different levels of the network. At this point, different classifiers may be applied, depending on the specific target application or control task one aims to accomplish [137]. The fused information provides a more comprehensive understanding of the user intent and context, enabling more intuitive and precise control, as presented in [138] with the integration of audio-visual feedback into a BCI-based control of a humanoid robot. For example, a BCI command to control a prosthetic limb could be validated and refined by RGB-D data showing the current user orientation and body posture. A similar solution was presented by Zhang et al. in [139], where an intelligent BCI system switch, based on a deep learning object detection algorithm (YOLOv4), was designed to improve the level of user interaction. The performance of the brain-controlled prosthetic hand, and its practical effectiveness, were tested by experiments in real scenarios. Real time detection of the prosthetic hand with confidence higher than 96% was achieved by the YOLOv4 tiny model, and the system was able to support the execution of four types of daily life tasks in real conditions, with acceptable execution time.

RGB-D sensing and BCI integration would consequently represent a powerful application of multi-modal fusion, where the complementary strengths of each technology are leveraged for an advanced form of adaptive human–machine collaboration. A dynamic intelligence loop, where the system continuously infers user intent from BCI signals and contextualizes it with information from RGB-D data, would create a full user-in-context understanding, moving AAL systems from passive monitoring to truly intelligent, adaptive, and user-centric assistance. A recent paper by Bellicha et al. [140] describes an assist-as-needed sensor-based shared control (SC) method, relying on the blending of BCI and depth-sensor-based control, which is able to reduce the time needed for the user to perform tasks and to avoid unwanted actions of a robotic manipulator. However, in this case, though, a cortical implant featuring 64 electrodes has been employed. Table 9 summarizes the relevant studies mentioned above, with regard to their objective, methodology, test population and main results.

### 5.2. Opportunities of RGB-D and BCI Fusion

Once integrated, RGB-D and BCI systems may operate under shared control or hybrid control paradigms [127]. In these models, the BCI-derived user intent, such as a high-level command or a desired direction of motion, is intelligently blended with sensor-based environmental awareness from RGB-D cameras. This would enable full adaptation of the assistance levels, ensuring that technology augments, rather than replaces, human action, which is crucial for user empowerment and long-term acceptance in AAL.

**Figure 5:**
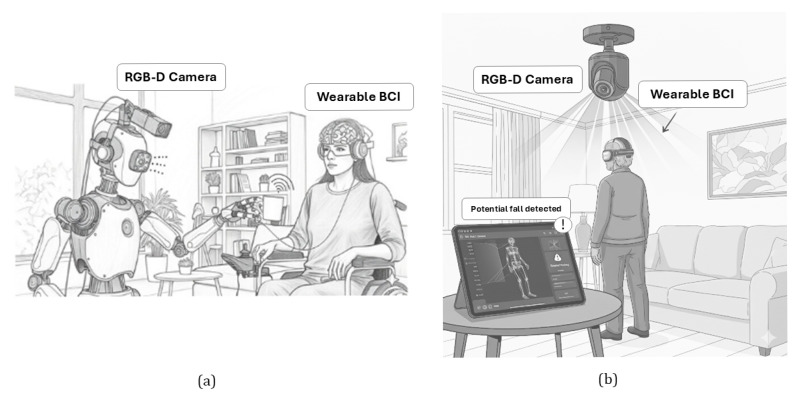
Examples of envisioned AAL scenarios supported by RGB-D camera and BCI integration: (**a**) adaptive robotic assistance, (**b**) personalized fall prevention.

The synergistic integration of RGB-D cameras and non-intrusive BCIs could unlock a new generation of AAL applications, moving beyond conventional monitoring to provide more comprehensive, personalized, and proactive support. These novel applications would represent a significant leap in AAL, transforming solutions to empower people with greater independence, safety, and a higher quality of life. The fusion would enable systems that not only understand what is happening (RGB-D) but also why (user intent from BCI) and how to best intervene (environmental context joint user state), leading to highly personalized, proactive, and effective support. New possibilities for maintaining dignity and autonomy in aging would be enabled in domains such as adaptive robotic assistance (with assistive robots capable of performing activities of daily living with unprecedented precision and user-driven intent), personalized fall prevention and response (monitoring the user cognitive state through BCIs could enable fall prediction and proactive risk mitigation), and intuitive smart home control (with contextual information provided by RGB-D cameras and natural interaction supported by BCIs). Two possible examples are shown in Figure 5.

### 5.3. Challenges in RGB-D and BCI Integration

Despite its promises, the integration of RGB-D cameras and BCIs also introduces significant challenges that must be addressed. They are similar to those pointed out by papers proposing multi-modal sensing applied to HAR, based on the integration of RGB-D cameras, wearable devices and inertial sensors [28], or RGB-D cameras and radar sensors [135], and several considerations are needed regarding multi-modal systems. First, real-time fusion of high-resolution video/depth data with high-dimensional EEG data is computationally demanding, and multi-modal sensor integration demands computational power and real-time processing capabilities. Indeed, RGB-D cameras generate high-resolution RGB, depth and skeleton data, while non-invasive BCIs produce noisy real-time brain signals [141], especially in uncontrolled scenarios such as home living environments. As highlighted by Karim et al. [29], computational requirements of HAR systems vary significantly across diverse applications. Low latency and almost real-time processing are typically needed by systems designed for healthcare monitoring and smart homes. Modern approaches based on data mining and analytics may ensure robustness against noise and occlusions and computational efficiency as required by real-world AAL systems [142].

Additionally, there is a need for improved hardware and signal processing to make wearable BCIs more practical in daily living. Current EEG-based BCIs can be cumbersome or intrusive, and their signals are prone to artifacts from motion and muscle activity. On the one hand, miniaturized depth sensors have already been proposed and their performance evaluated [143]. On the other hand, innovations such as miniaturized, wireless BCI headsets with better signal quality are needed to ensure the system is both unobtrusive and reliable during routine activities. Progresses has been recently reported in the design and experimental validation of a minimally obtrusive platform for EEG recordings by a network of miniaturized wireless electrodes [144], which paves the way to future improvements and optimized design. Indeed, without reducing the burden of wearing and maintaining BCI devices, users may be reluctant to use them continuously, undermining the system effectiveness.

Privacy implications and data security issues cannot be overlooked, with both technologies (RGB-D cameras and BCIs) being deployed in intimate private spaces and used for monitoring sensitive activities. BCIs may raise even more profound privacy concerns than RGB-D cameras, as they collect exceptionally sensitive and personal neural data, capable of revealing intimate information about individual thoughts, emotions, and subconscious states [145]. While approaches to ensure security and privacy when using BCIs have been experimentally validated, as detailed in [146], new solutions are being proposed for the deployment of trusted BCI applications outside of controlled laboratory settings [147]. Mitigating privacy concerns through personalized, adaptive, and transparent design is essential to overcome psychological barriers, build long-term trust, and ensure the sustained engagement and effectiveness of these advanced assistive technologies.

User acceptance, comfort, and technology illiteracy are paramount factors for long-term successful deployment and widespread adoption of AAL systems [148,149]. Although the acceptance of camera-based monitoring has been analyzed in many studies in the literature [150], additional investigations would probably be needed for AAL systems that integrate BCIs, for which illiteracy refers to the inability of some individuals to effectively use BCIs despite their best efforts, thus preventing widespread adoption [151]. Variability in user capability means the system must be adaptable and cannot assume that one size fits all in terms of brain control performance. User-centric design and training will be necessary to mitigate these adoption barriers. Finally, properly structured ethical and legal frameworks for neural and visual (i.e., RGB-D) data will be of crucial importance to ensure responsible innovation and compliance, but also to build public trust, mitigate potential harms (e.g., discrimination, manipulation), and defend fundamental human rights, such as cognitive freedom [152,153]. In fact, current regulations for home monitoring and medical devices may not fully cover the nuanced risks coupled with the processing of neurophysiological data. Issues of consent, data ownership, and potential misuse (for instance, using brain data for purposes beyond the stated intent) are not yet fully resolved [154]. Without well-defined governance, there is a risk that user cognitive privacy or agency could be compromised. Developing guidelines to protect user neural data and uphold rights like cognitive freedom is therefore essential as neurotechnology becomes more pervasive in the living environment.

### 5.4. Open Issues in RGB-D and BCI Integration

Looking ahead, future research should focus on several key directions to fully realize the envisioned benefits of RGB-D and BCI integration for HAR:

- *Lightweight multi-modal fusion algorithms:* There is a pressing need to develop more efficient data fusion and ML/DL algorithms to handle multi-stream RGB-D and EEG data in real time, without excessive computational load. In particular, this can include the design of optimized and efficient feature extraction methodologies [155], that, however, must take into account the heterogeneous nature of the input data, i.e., images and biosignal time series. In this case, adopting multi-domain features and advanced methodologies for feature selection and information fusion [156] represent attractive possibilities for the optimization of the feature extraction pipeline, reducing the computational burden of this processing step. However, it is worth noticing that RGB-D images require the major part of the computational resources in terms of data processing. Thus, other viable solutions can be found by considering techniques for compressing images and removing non-necessary or redundant information [99]. On the other hand, there is also the need for exploring computational solutions, optimized neural networks, and advanced signal processing techniques to ensure the system can run continuously in a home environment (potentially on embedded hardware) without sacrificing accuracy. This can be achieved by leveraging tiny learning models specifically designed for mobile or low-resource devices [157], and by relying on the growing computational power of newer devices. Another approach to deal with these issues is to transfer part of the computational demands to the cloud or shared services, or to adopt edge computing solutions within an optimized framework [158].
- *Robustness in unconstrained environments:* To be practical in daily living, HAR systems must be resilient to the messy, unpredictable nature of real homes. Future studies should emphasize improving the robustness and accuracy of activity recognition under real-world conditions, such as varying lighting, background clutter, the presence of multiple subjects, or user movement, as well as coping with EEG noise from muscle activity or electrical interference. The latter can be addressed by relying on lightweight but robust recently proposed algorithmic solutions [159]. This may involve creating large and diverse datasets for training, using adaptive algorithms that can learn from a user routine, or integrating additional context (e.g., time of day, habitual patterns) to reduce false detections. This point represents one of the major issues for the deployment of image-based, hybrid HAR solutions within real environments. Indeed, as outlined also in previous sections, in practice it is not possible to enforce the presence of a single user within a certain environment. In practical applications, it is also likely that different users would perform different activities to be recognized. To address the complexities imposed by a multi-person scenario, the usage of multiple cameras can be a viable solution [103], while the recognition of multiple activities performed at the same time would require the usage of tailored and specific processing procedures [104]. Real environments, with their complex backgrounds, can often lead to occlusion problems that can be faced by relying on multiple-angle views of the same scene [160]. Variable lighting conditions, which can heavily affect the outcome of HAR under consideration, could also be successfully handled through specific post-processing procedures, nowadays also tailored to limited computational resources, thus reducing their impact on the demands of the overall recognition architecture [161].
- *More user-friendly BCI devices:* Engineering advances are needed to design BCIs that are comfortable, unobtrusive, and easy to operate for non-expert users. This could mean wireless, miniaturized EEG systems with dry electrodes (avoiding lengthy setup), longer battery life, and auto-calibration features. Improving signal quality through better sensors or algorithms (to filter out artifacts) will also enhance reliability. By making the BCI hardware invisible and hassle-free, users are more likely to wear it regularly, which is crucial for continuous HAR in AAL. In particular, an attractive solution for the envisioned framework can be the usage of in-ear EEG-based BCI [162], which has been conceived in order to offer an alternative way for recording brain activity by using probes placed on the ear and in the ear canal. Even though the EEG acquired with this kind of sensing technology does not fully meet all the characteristics provided by scalp EEG, in-ear technology represents a suitable compromise for developing lightweight, unobtrusive, and comfortable BCI applications. Furthermore, in-ear EEG systems have also been integrated within large body-area networks for health monitoring [163], thus pointing out the combination of this kind of technology with RGB-D systems as an actual possible solution for RGB-D and BCI fusion.
- *User acceptance and usability studies:* Going forward, researchers should extensively study how target user groups (e.g., older adults, people with disabilities) perceive and interact with these integrated systems. Early involvement of end-users through participatory design can identify usability pain points and preferences, ensuring the solutions truly meet user needs. In fact, further investigations into user acceptance of BCI-augmented AAL systems are needed [164], as prior work has mainly examined acceptance of camera-based monitoring alone. Understanding factors that influence trust, such as system transparency, feedback provided to the user, and perceived benefits, will be vital. Strategies to improve acceptance might include personalized adaptation (tuning the system responses to individual comfort levels), training and onboarding programs to help users get comfortable with BCIs, and integrating privacy-respecting options (for example, allowing users to easily pause or control data collection). User acceptance and usability could be further enhanced by developing user-independent architectures for BCI applications in order to avoid the need for retraining the model when used on unseen, new individuals. For achieving this objective, some promising solutions are currently available, allowing also to address the cross-session issue [165]. By applying such kind of processing on EEG data, it would also be possible to lower computational demands and time needed for calibration, since data from other users or from different sessions could be used in the initialization phase, enhancing also the usability and acceptability of the BCI usage in a real-life scenario.
- *Privacy protection and ethical frameworks:* In tandem with technical improvements, there must be a concerted effort to establish comprehensive ethical and legal guidelines for deploying these technologies in private homes. Future work should engage ethicists, legal experts, and policymakers to develop standards for data handling that ensure strict privacy, security, and user consent. This includes determining how neural and video data should be stored and used and setting limits to prevent any form of data abuse [153]. Researchers have noted that mitigating privacy concerns through personalized, transparent system design is essential to overcome user barriers and build long-term trust, and that properly structured frameworks will be crucial for responsible innovation in this domain [154]. By embedding ethical considerations into the design (such as on-device data processing to keep raw data private or providing users with clear control over their information), developers can foster greater user confidence and societal acceptance of these advanced AAL solutions [166]. It is worth noticing that for addressing privacy issues, the usage of skeleton data extracted from RGB-D images appears convenient, since this kind of representation modality discards any user identity information, also making the background scene not identifiable. However, as discussed also in the previous sections, the usage of a multi-modal approach, where skeleton and RGB-D modalities are combined, provides significant advantages in terms of HAR results. Also, multi-modal schemes proved to be less vulnerable under adversarial attack, thus providing more robust applications in terms of safety and privacy concerns [87,167]. Therefore, balancing ethical concerns and technical outcomes still remains an open issue that should be evaluated depending on settings, environments, target users, and desired results for each specific HAR application.

## 6. Conclusions

Starting from the overview about the use of RGB-D cameras and non-invasive BCIs as single sensing modalities in HAR for AAL, this perspective shed light on the opportunities, challenges, and potential disruptive innovations that could stem from an integrated dual-perception framework, underpinning the transformative potential of RGB-D+BCI systems for AAL. Fusing environmental and cognitive data streams, such systems can infer not only what a user is performing, but also why and how they are performing it. This kind of enriched context is simply unattainable with vision or BCI alone. It is precisely the integration of these two dimensions that enables the development of innovative solutions to improve quality of life in AAL, aligning with the core goal of AAL technologies to support independent living and well-being. In essence, the system becomes both situation- and user-aware, which represents a paradigm shift in HAR: from reactive monitoring of observable actions to proactive understanding of user needs and states, enabling possible anticipatory actions. This approach promises to enhance user independence, safety, and quality of life, which are core goals of AAL, by allowing proactive and situation-specific interventions.

Integrating RGB-D environmental perception with non-invasive BCI-derived insight into the user state constitutes a powerful dual-perspective approach to HAR. This perspective paper has articulated how such synergy offers a more complete picture of daily activities, effectively bridging the gap between external actions and internal experiences of a person. By focusing on the combined analysis of what is happening around the user and what is happening within the user’s mind, future HAR systems can become significantly more context-aware, personalized, and responsive. The concept of integrated dual perception highlighted here is not only a novel contribution to the field of HAR but also a promising foundation for next-generation AAL technologies that aim to improve safety, independence, and quality of life for aging or vulnerable populations.

Continued research and development along the lines discussed, from algorithmic innovations to privacy-preserving and human-centered design, will yield intelligent living environments capable of not just monitoring their inhabitants but truly understanding and anticipating their needs. This integration of external and internal sensing thus serves as a key enabler in achieving the full potential of active and assisted living. In fact, a key insight that arises from this envisioned dual-modality approach is that the fusion of these two dimensions, namely environmental context and user state, provides a far more holistic understanding of human activities than either modality alone. Their integration enables context-aware interpretations of behavior that were not previously possible.

## Figures and Tables

**Table 1:** Common devices for main ADLs.

Activity	Vision-Based Devices	Wearable Devices
Body movements	RGB-D cameras, video cameras, thermal cameras	IMUs, electromyography sensors, photoplethysmogram sensors, skin conductance sensors
Hand gestures (including eating and drinking)	RGB-D cameras, video cameras, thermal cameras	IMUs, electromyography sensors
Sleeping	RGB-D cameras	photoplethysmogram sensors, skin conductance sensors
Standing/falling	RGB-D cameras, video cameras	IMUs, electromyography sensors
Sitting with mental occupation (including studying and working)	RGB-D cameras, video cameras	BCIs, photoplethysmogram sensors, skin conductance sensors

**Table 2:** Advantages and disadvantages of RGB-D cameras.

Advantages	Disadvantages
reduced size	sensitivity to thermal noise and lighting variations
non-invasive monitoring	high storage resources for onboard data processing
costs and performance trade-off depending on sensing principle	wide occupied bandwidth for remote data processing

**Table 3:** Comparison of sensing principle among commercial RGB-D cameras.

Manufacturer	Commercial Name	Depth-Sensing Principle
Intel	D400 series	stereo vision
RealSense	L515	LiDAR technology
Microsoft	Kinect v1	structured light (IR projection)
	Kinect v2	ToF
Orbbec	Astra Pro	ToF (nIR)
Others	Structure IO	structured light (IR projection)
	Asus Xtion Pro	structured light (IR projection)

**Table 5:** Comparison of works using uni-modal approach for HAR from RGB-D images. Classification accuracy values are from original studies.

Work	Architecture	Input Data	Outcomes	Key Novelty Point
Park et al. [47]	LSTM	Skeleton data	99.5% accuracy on MSRC-12 [47]	Leveraging time sequential encoding of activity features
Mitsuzumi et al. [56]	GCN	Skeleton data	67.4% accuracy on NTU-RGB+ D60 [60], 57.7% on NTU-RGB+ D120 [61]	Introducing a subject-agnostic domain adaptation, randomizing the frequency phase of motion data, leaving amplitude unchanged
Karthika et al. [58]	A stacked ensemble model made by SBGTGCN, 2DCNN+2P-LSTM, and 3DCNN+XGBoost	Skeleton data	97.9% accuracy on NTU-RGB+ D60 [60], 97.2% on NTU-RGB+ D120 [61], 97.5% on Kinetics-700-2020 [62], 95.2% on MA-52 [63]	Deriving 3D skeletal points by Gaussian RBF, and designing a stacked ensemble model that integrates multiple base learners and a meta-learner
Chen et al. [72]	GCN and Transformer model	Skeleton data	92.7% accuracy on NTU-RGB+ D60 [60], 86.8% on NTU-RGB+ D120 [61], 39.0% on Kinetic-Skeleton [80]	Design of GCN and Transformer parallel stream for extracting local and global features as topological structures and inter-joints connections
Wu et al. [53]	2D CNN	Video frames	91.9% accuracy on NTU-RGB+ D60 [60], 92.2% on Kinetics-700-2020 [62], 96.9% on UFC101 [68], 73.7% on HMDB51 [81], 77.9% on STH-STH [82]	Design of a multi-level channel attention excitation module to retrieve highly discriminative video feature representation
Zong et al. [54]	Hybrid CNN-LSTM	Video frames	94.7% accuracy on UFC101 [68], 67.2% on HMDB51 [81], 68.7% on Kinetics-700-2020 [62]	Design of a four-stream network, based on spatial and temporal saliency detection
Hussain et al. [55]	Hybrid CNN-LSTM	Video frames	98.7% accuracy on UFC101 [68], 80.3% on HMDB51 [81], 98.9% on UCF50 [83], 98.9% on YouTube Action [84]	Design of a dynamic attention fusion unit and a temporal-spatial fusion network to extract human-centric features from temporal, spatial, and behavioral dependencies
Li et al. [64]	Spatial-temporal mixed module MLP	Video frames	93.6% accuracy on CAD-120 [65], 86.1% on STH-STH V1 [82]	Introduction of text features for human-object interaction and of supervised natural language learning for augmentation of visual feature representation
Elnady and Abdelmunim [67]	LSTM	Video frames	96.0% accuracy on UFC101 [68], 99.0% on KTH [69], 98.0% on IXMAS [71], 100% on WEIZMANN [70]	Combining YOLO and LSTM to integrate highly discriminative features from individual frames and sequential temporal dynamics of motion

2P-LSTM: two-part LSTM; GCN: graph convolutional network; MLPs: multilayer perceptrons; SBGTGCN: spatial bidirectional gated temporal graph convolutional network with attention; RBF: radial basis function.

**Table 6:** Comparison of works using a multi-modal approach for HAR from RGB-D images. Classification accuracy values are from original studies.

Work	Architecture	Input Data	Outcomes	Key Novelty Point
Bruce et al. [88]	GCN and attention mechanism	Skeleton and RGB data	93.9% accuracy on NTU-RGB+ D60 [60], 90.5% on NTU-RGB+ D120 [61], 96.3% on PKU-MMD [74], 77.5% on Toyota Smarthome [79], 93.5% on Northwestern-UCLA Multiview [77]	Multi-modal network that fuses skeleton and RGB complementary information, using GCN to learn attention weights from skeleton that are then shared with the RGB network
Kumar and Kumar [89]	Hybrid CNN-BiLSTM	Depth, RGB, and skeleton data	96.2% accuracy on UTD-MHAD [78]	Design of a multi-view, multi-modal framework, where RGB, depth, and skeleton data are separately processed by 5S-CNN and BiLSTM networks, and the outputs are fused by a weighted product model
Batool et al. [90]	CNNGRU	RGB, depth, and inertial data	97.9% accuracy on HWU-USP [76], 97.9% on Berkeley-MHAD [75], 96.6% on NTU-RGB+ D60 [60], 95.9% on NTU-RGB+ D120 [61], 97.9% on UTD-MHAD [78]	Introducing novel features extracted from RGB, depth, and inertial data, where redundant information is profiled out by a genetic algorithm
Liu et al. [95]	Hybrid GCN-CNN	Skeleton and RGB-D video	94.8% accuracy on NTU-RGB+ D60 [60], 97.0% on PKU-MMD [74], 93.7% Northwestern-UCLA Multiview [77]	Introducing a semantic-assisted framework where text modality is added to RGB and skeleton, with a visual-language module with contrastive language-image pretraining
Song et al. [96]	Hybrid CNN-LSTM	Skeleton and RGB-D video	93.8% accuracy on NTU-RGB+ D60 [60], 76.9% on MSR-3D [85], 85.7% on UCF101 [68], 64.8% on JHMDB [86]	Introducing a modality compensation network for fusing multiple representation modalities, including adaptation schemes for narrowing the distance between different modalities distributions
Liu et al. [97]	Hybrid GCN-CNN-RNN	Skeleton and RGB-D video	94.3% accuracy on NTU-RGB+ D60 [60], 96.8% on PKU-MMD [74], 93.9% on Northwestern-UCLA Multiview [77]	Designing of a temporal cues enhancement module for improving temporal modeling from RGB modality

CNNGRU: convolutional neural network–gated recurrent unit; GCN: graph convolutional network; RNN: recurrent neural network.

**Table 7:** Primary applications of main non-invasive BCIs.

Company/Product	Brief Description	Applications
Emotiv (EPOC X, MN8 EEG)	Wireless EEG headsets	Neuroscience research, neurofeedback, gaming, cognitive wellness monitoring
OpenBCI (Ultracortex Mark IV, Cyton, Galea)	Open-source BCI hardware and software	Research, development, hobbyist projects, gaming, AR/VR/XR with integrated biosensing
g.tec (Unicorn Brain Interface, g.HIamp PRO)	Complete BCI systems and components (amplifiers, EEG headsets)	Research, rehabilitation, clinical applications
NeuroMaker BCI	BCI kit for educational purposes	Learning about neuroscience, visualizing brainwaves, mind-controlled games
PiEEG	Low-cost EEG devices and BCI kits	Learning, research, development
Neurable	BCI technology for controlling digital objects	Gaming, augmented/virtual reality, thought control
NextMind (by Snap)	BCI technology for decoding neural activity	Controlling digital objects (acquired by Snap, focus on AR/VR)

**Table 8:** Advantages and disadvantages of BCI interfaces.

Advantages	Disadvantages
customized properties for users	required training phase on users
invasive/non-invasive mode depending on acquired signals	sensitivity to non-linearity and noise
signals monitored by multiple channels	non-stationary acquisition process

**Table 9:** Comparison of relevant studies adopting RGB-D cameras and BCI integration in AAL.

Study	Objective	Method	Population	Results
Pereira et al. [129]	A dynamic visual interface to navigate the indoor environment, which exploits RGB-D perception to improve BCI-based actuation of a wheelchair	RGB-D images and BCI signals, separately processed, are merged in a dynamic visual interface. User intent gathered by the P300 device is matched to bounding boxes detected on RGB-D images	5 participants (23–31 years old, 3 males, 2 females)	89.9% and 86.4% an average accuracy, respectively, for non-self-paced and self-paced selection of 30 predefined target events, with average effective symbol per minute (eSPM) of 4.8 and 4.7
Mezzina et al. [131]	A smart sensor system to implement a BCI-controlled human–robot interface for AAL	Direct communication path between the human brain and external actuator. BCI decodes user intention by a fast classifier; heterogeneous sensors (RGB-D cameras, sonar, and IR sensors) onboard a personal care robot are exploited to ensure correct actuation of user intent	4 participants (26 ± 1 years old)	84% accuracy in user intent classification—75% success rate in correct actuation execution
Ban et al. [132]	A multifunctional (10 actions) NAO v6 robot control system based on a multi-modal brain–machine interface (BMI) that fuses three signals: steady-state visual evoked potential (SSVEP), electrooculography (EOG), and gyroscope	Hybrid convolutional neural network—bidirectional—long short-term memory (CNN-Bi-LSTM) architecture based on attention modules, to extract temporal information from sequence data and enable classification	16 participants (19–25 years old, 8 males, 8 females)	93.78% accuracy in completion of complex tasks, by all participants—average response time of 3 s
Muñoz et al. [133]	A cost-effective rehabilitation system (named brain–kinect interface, BKI) based on videogames and multi-modal recordings of physiological signals (by a consumer-level EEG device + Kinect)	A gesture interaction module (using Kinect) and a BCI dynamically monitor physiological variables of patients while they are playing selected exergames for rehabilitation	Not specified for the proposed system validation—up to 700 patients involved in exergames only	Technical figures not reported—improvements in postural balance (+15% balance time increase) and range of motion (up to +18%)
Tidoni et al. [138]	Visual information and auditory feedback fused to improve the BCI-based remote control of a humanoid surrogate (HRP-2 robot) by people with sensorimotor disorders	An SSVEP BCI classifier decodes user intent that is associated with visually recognized objects captured by the embedded robot camera. Objects are paired with offline pre-defined tasks, triggered by the user SSVEP	14 healthy participants (25.8 ± 6.0 years old, 6 females) and 3 subjects who had suffered traumatic spinal cord injury (22–31 years old, 3 males)	Action-related feedback may improve subject information processing and decisions about when to start an action—no significant differences in task completion time and placing objects accuracy
Zhang et al. [139]	To improve the interaction and practical application of a prosthetic hand with a BCI system, by integrating augmented reality (AR) technology	An asynchronous pattern recognition algorithm, combining center extended canonical correlation analysis and support vector machine (Center-ECCA-SVM), is proposed, together with a deep learning object detection algorithm (YOLOv4) to improve the level of user interaction in 8 pre-defined control modes	12 participants	96.7% average stimulus pattern recognition accuracy—96.4% confidence in prosthetic hand real-time detection by YOLOv4 tiny model
Bellicha et al. [140]	An assist-as-needed sensor-based shared control (SC) method relying on the blending of BCI and depth-sensor-based control targeting mobility and manipulation needs in home settings	Shared control (SC): control shared between user command (retrieved from a control interface) and sensor-based control by features detected by robot-embedded sensors	A quadriplegic patient	Time to perform tasks and number of changes in mental tasks were reduced, unwanted actions avoided

## Data Availability

No new data were created or analyzed in this study. Data sharing is not applicable to this article.
